# Theoretical, Clinical, and Rehabilitative Aspects of Anosognosia an Extended Editorial

**DOI:** 10.3390/brainsci16020184

**Published:** 2026-02-02

**Authors:** Guido Gainotti

**Affiliations:** Institute of Neurology, Università Cattolica del Sacro Cuore, Fondazione Policlinico A. Gemelli, Istituto di Ricovero e Cura a Carattere Scientifico, 00168 Rome, Italy; guido.gainotti@unicatt.it; Tel.: +39-06-30156435

## 1. Introduction

Anosognosia (from the ancient Greek—a-, “without,” nosos, “disease,” and gnōsis, “knowledge”) has been recognised as one of the most complex syndromes investigated from different theoretical and clinical perspectives in patients with brain damage since the beginning of the last century (see [1,2,3,4] for reviews). Many controversial aspects of this syndrome were raised by Babinski [5,6,7], who, in his pioneering observations of patients unaware of their hemiplegia, highlighted two main points: (a) anosognosia for hemiplegia is usually due to right hemisphere lesions; (b) this syndrome could be due not only to a (cognitive) lack of knowledge but also to a (motivated) denial of the hemiplegia, providing a protective effect. The complexity of this condition was exacerbated by fact that the term “anosognosia” was chosen by Babinski [5] to denote this phenomenon, who has rightly pointed out that this general term could refer not only to one’s unawareness of one’s hemiplegia but also to that of many other disabilities. Before Babinski, several authors had shown that brain-damaged patients may be unaware of certain kinds of impairment. Among these are sensory defects such as cortical blindness or hemianopia (e.g., Anton, [8]), language disorders (e.g., [9,10]), neglect of the space to one’s left (e.g., [11,12]), memory disorders (e.g., [13,14]), Alzheimer’s disease (e.g., [15]), and many other infirmities. More recently, some authors (e.g., [16]) have stressed the fact that anosognosia can be selective; a patient with multiple disabilities may be unaware of one handicap while appearing fully aware of the others. This phenomenon suggests that anosognosia may, at least in part, be due to the disruption of domain-specific awareness mechanisms, and that brain damage can selectively impact the self-monitoring process of specific physical or cognitive functions rather than affecting a general control function subserved by the frontal lobes. Another important source of diagnostic difficulty stems from the acknowledgement that anosognosia is not a strongly unitary phenomenon; a diagnosis of disease unawareness can be made under some exam conditions but not others. A distinction has been made by Crosson et al. [17] and Toglia and Kirk [18] between three different levels of anosognosia. In the most stable and severe (“*intellectual*”) level [17], patients are unable to understand that they have a specific disability and that, therefore, they will not be able to perform a related task. In the second (“*emergent*”) level, patients are initially unaware of their disability but can recognise the existence of an infirmity when a related problem occurs. In the mildest (“*anticipatory*”) level, the patient is not fully aware of their disability but can anticipate that difficulties might occur as a consequence of certain deficits and that these difficulties can be reduced by specific compensations. These distinctions are important not only from a theoretical but also from a prognostic and rehabilitative perspective. They must be incorporated into theoretical models of anosognosia and must be considered in the rehabilitation of patients who are unaware of their infirmity.

## 2. Specific Problems Addressed in this Special Issue

Most of these theoretical and rehabilitative problems have been considered from several different perspectives in the papers published in this Special Issue of *Brain Sciences* and will shortly be discussed below.

The first two problems that will be considered were anticipated by Babinski and concern, respectively, (a) the relations between anosognosia and lesion laterality, and (b) the role of emotion as a factor involved in the development of anosognosia and in its rehabilitation. The third problem (which is very important from a clinical point of view) concerns the rehabilitation strategies proposed for the treatment of anosognosia.

### 2.1. The Relations Between Anosognosia and Lesion Laterality

In the conclusion of his landmark contribution to the study of anosognosia for hemiplegia (AH), Babinski [5], having described two patients with right hemisphere (RH) stroke who were unaware of their hemiplegia, wondered whether AH might be specific to right hemisphere lesions. Ascribing to the RH a prevalent role in the development of AH was, however, complicated by confounding effects arising from the presence of aphasia in patients with left hemisphere lesions and from unilateral spatial neglect in many anosognosic patients with RH stroke. These objections have recently been supported by results obtained by Cocchini et al. [19], who showed that the frequency of AH may have been underestimated in left-brain-damaged patients for certain methodological reasons and that AH may also be associated with left hemisphere lesions. The concomitant hypothesis, assuming that AH may be the by-product of ipsilateral neglect for personal and extra-personal space, was also supported by the clinical observations of authors who found a strong association between AH and unilateral neglect (see the reviews in Vuilleumier [20]). However, results obtained in more controlled conditions, such as via studies in which AH was evaluated during intracarotid barbiturate injection (e.g., [21]), have allowed us to conclude that the apparently greater role of the RH in the development of AH is not simply due to confounding variables, and by studies showing that AH is not a consequence of spatial neglect, because patients with AH and left neglect still deny their motor weakness when the affected limbs are shown in the intact right side of space (see, e.g., [22]).

In this Special Issue, the problem of the relationship between anosognosia and lesion laterality has been considered from various theoretical and clinical perfectives, focusing both on anosognosia for hemiplegia and on other, less frequent forms of disease unawareness. In particular, Langer and Bogousslavsky [23], in their general discussion of unawareness and denial mechanisms, have proposed that anosognosia may be due to belief distortions that conflict with reality in an effort of making sense of personal limitations, and that these beliefs distortions may be predominantly associated with right hemisphere damage. According to this perspective, beliefs and one’s failure or difficulty to update them are not due to purely cognitive factors but can be influenced by strong motivational factors, and the difficulty faced in updating pre-existing beliefs could play a pivotal role in the development of anosognosia. In a much more clinically oriented approach to the motor and non-motor aspects of anosognosia for hemiplegia, Beccherle et al. [24] acknowledge that AH is a complex, multifaceted phenomenon and that the different forms in which it manifests create difficulties for both clinicians and researchers. These authors, therefore, remain very cautious with respect to the problem of the relationship between AH and lesion laterality because they acknowledge that responses to this question will depend on the methods used for assessment. Dissociations can be found, for example, between situations in which patients explicitly (verbally) deny their paralysis and situations in which the same patients act as if they could, implicitly, know that they cannot move the paralysed body part. Another different approach to the problem of the relationship between anosognosia and lesion laterality has been taken by Luzzi et al. [25], who have investigated poor awareness of familiar person recognition disorders in patients with right and left variants of semantic dementia (SD). As far as we know, only Young et al. [26] had previously reported a single case study of a patient (SP) who had shown unawareness of impaired face recognition after a subarachnoid haemorrhage from a right middle cerebral artery aneurysm. Luzzi et al.’s paper [25] investigated this issue more thoroughly, building on the results of previous studies (e.g., [27,28,29]) which had shown (a) that familiar person recognition disorders via the face and voice are frequently observed in patients with the right variant of SD, and (b) that these disorders are mainly due to a disruption in the automatic generation of the corresponding feelings of familiarity underpinned by the right anterior temporal lobe (RATL). An association between name recognition disorders and disrupted “familiarity feelings” (underpinned by the left ATL), had, on the contrary, been previously found in patients with a left variant of SD [29]. This investigation therefore sought to determine if the prevalence of anosognosia in right-brain-damaged patients is greater for tasks in which the right hemisphere plays a dominant role and if this prevalence is, in part, due to the automatic processing mechanisms typical of this hemisphere. The results of the study in question showed that the right hemisphere plays a critical role in the generation of anosognosia because unawareness of person recognition disorders was significantly greater in patients with the right variant of SD for face recognition (in which the right hemisphere plays a dominant role) and because this prevalence was mainly due to the defective generation of feelings of familiarity typical of this variant of SD.

### 2.2. The Role of Emotion as a Factor in the Generation of Anosognosia and in Its Rehabilitation

As already noted in the introduction, the presence of an emotional component in patients unaware of their hemiplegia has also been highlighted by Babinski [7], who made the observation that some of his patients had, for many years, been very afraid of the motor impairment that they now apparently ignored. This mismatch between previous anxious expectancies and present lack of concern suggested that (at least in some patients) the subject’s apparent unawareness of the illness was influenced by motivational factors. The idea that anosognosia may not necessarily be due to defective awareness but must sometimes be considered an extreme form of adaptation to stress has more recently been developed by Weinstein and Kahn [30], who coined the term “Denial of Illness,” to define a “form of social behavior in which the patient by an altered mode of interaction in the environment adapts to the stress of his disability.”

In this Special Issue, the problem of the relationship between emotional and motivational factors and apparent unawareness of a disability has been considered from theoretical, clinical, and rehabilitative perspectives. From the theoretical perspective, Langer et al. stressed the fact that beliefs can be influenced by emotional and motivational factors. For these reasons, some denial just after a stroke could be adaptive, protecting the patient from being overburdened even if, at a later time, the same behaviour may become maladaptive [31], impeding functional rehabilitation progress. Langer et al. also note that the prevalence in right-brain-damaged patients of both anosognosia and important emotional disorders (e.g., [32,33,34]) raises the problem of the relationship between motivated denial and emotional factors in the context of hemispheric asymmetries. Similar positions are reached in this Special Issue by Beccherle et al., who, drawing on their careful clinical observations, acknowledge that in clinical practice, it is common to see individuals who seem to completely reject the idea of being paralysed. These patients usually recognise other minor disorders but deny their motor deficits, often using implausible excuses. Signals of an awareness that is not being openly expressed include be the presence of fluctuations in patients’ responses, as well as with behaviours that imply some knowledge of their paralysis.

From the perspective of rehabilitation, both Langer et al. and Beccherle et al. acknowledge that persistent unawareness or denial of a disability can impede the progress of functional rehabilitation. Furthermore, Beccherle et al. [24] consider whether both positive and negative emotional inductions (e.g., comments such as “Well performed” or “This is wrong”) can modulate the degree of anosognosia, and they conclude that only negative emotional inductions result in a significant improvement in motor awareness, whereas no effects are found with positive emotional induction (e.g., [35]). Another interesting observation on the role played by emotional disorders in functional rehabilitation progress in AH patients has been reported in this Special Issue by Bandiera et al. [36], who conducted a study on the relationships between emotional status, metacognitive self-awareness, and functional disability level after an acquired brain injury. These authors showed that patients with severe acquired brain injury who recover from impaired self-awareness may present with anxiety and/or depression as adaptive reactions to brain damage and related functional disabilities. These authors conclude that the neurorehabilitation of individuals with moderate-to-severe acquired brain injury should address the complex interactions between impaired self-awareness and related anxious and depressive conditions.

### 2.3. Rehabilitation Strategies Proposed for the Treatment of Anosognosia

Some important behavioural patterns that could play a role in the treatment of anosognosia have already been mentioned in previous sections. We refer, here, to the use of emotional anosognosia modulation strategies through positive or negative comments on the patient’s actions (e.g., [37,38]), as well as to controlling the interactions observed during the rehabilitation process between impaired self-awareness and anxious and depressive reactions to these changes (e.g., [39,40]). A more complete approach to this problem can be found in the systematic review of the treatment of anosognosia for hemiplegia in stroke performed by Kim et al. [41]. The authors showed that interventions emphasising motor intention monitoring, namely, recalibrating the internal self-models by which individuals anticipate and verify their own actions, offered more promising and sustained therapeutic effects, and that error correction and self-observation were more consistently associated with durable improvements than neglect-based spatial interventions. As a matter of fact, studies employing error-based learning paradigms [42] by which patients compare discrepancies between intended actions and actual motor outcomes have consistently demonstrated a progressive recovery of motor awareness over time. Analogously, interventions based on video feedback and self-observation [43,44] facilitated improvements by allowing patients to adopt a third-person perspective on their own motor performance, thus bypassing immediate and potentially impaired first-person monitoring circuits.

## 3. Concluding Remarks

Taken together, the papers included in this Special Issue indicate that the most significant progress made in the study of anosognosia in recent years concerns data acquired through the systematic clinical observation and treatment of anosognosic patients, and that these advances concern not only rehabilitation but also the mechanisms of disease unawareness. Thus, the proposal that the prevalence of AH in patients with RH stroke is not due to the presence of ipsilateral neglect in these patients is consistent with both clinical and rehabilitation observations. Thus, Beccherle et al.’s [24] clinical observation that anosognosia is not a “consistent” clinical condition (as unilateral neglect is) is in agreement with Kim et al.’s [41] conclusion that treatments stressing error correction and self-observation are more frequently associated with durable improvements in motor awareness than neglect-based spatial interventions. On the other hand, a careful observation of what happens during these treatments has shown that strategies of error correction and self-observation do not necessarily lead to a consistent and progressive decrease in anosognosia; they can lead to symptom fluctuations and to incongruent and inconsistent responses. Very illustrative of these situations is the case reported by Allum et al. [45] of a patient for whom video feedback therapy had been successful in improving his awareness of hemiplegia, but who, having been extremely distressed by this “paradigm shift,” sought, after several iterations, to avoid further involvement in this form of treatment. Taken together, these clinical and rehabilitative observations support the motivational approach and, in particular, the suggestion of Turnbull and Solms [46] that the lesions under consideration may damage a right-sided emotion-regulation system, leading patients to deny their deficits because they experience overwhelming difficulty in tolerating aversive emotional states such as those related to severe motor impairment. The papers included in this Special Issue clearly show that the study of the mechanisms underlying one’s unawareness of hemiplegia and other disabilities remains a fascinating and attractive subject.
